# Polyvinylsulfonic acid: A Low-cost RNase inhibitor for enhanced RNA preservation and cell-free protein translation

**DOI:** 10.1080/21655979.2017.1313648

**Published:** 2017-06-29

**Authors:** Conner C. Earl, Mark T. Smith, Richard A. Lease, Bradley C. Bundy

**Affiliations:** a Department of Chemical Engineering, Brigham Young University, Provo, UT, USA; b Department of Chemical and Biomolecular Engineering, The Ohio State University, Columbus OH USA

**Keywords:** in vitro transcription and translation, nuclease inhibition, polyvinyl sulfonic acid, RNase inhibitor, RNA storage

## Abstract

The effectiveness and economics of polyvinyl sulfonic acid (PVSA) as a ribonuclease inhibitor for *in vitro* systems is reported. PVSA was shown to inhibit RNA cleavage in the presence of RNase A as well as in the presence of *Escherichia coli* lysate, suggesting that PVSA can act as a broader ribonuclease inhibitor. In addition, PVSA was shown to improve the integrity of mRNA transcripts by up to 5-fold *in vitro* as measured by their translational viability. Improved preservation of mRNA transcripts in the presence of PVSA under common RNA storage conditions is also reported. A cost comparison with commercially available RNAse inhibitors indicates the economic practicality of PVSA which is approximately 1,700 times less expensive than commonly used ribonuclease inhibitors. PVSA can also be separated from RNA by alcohol precipitation for applications that may be sensitive to the presence of PVSA.

## Introduction

RNA plays a vital role in myriad biologic processes including protein translation, gene regulation, and gene expression. Beyond its natural functions, RNA has been engineered for diverse applications including therapeutics development, medical diagnostics, and protein engineering.^1-4^ In particular, messenger RNA (mRNA) is naturally prone to rapid degradation by ubiquitous Ribonucleases (RNases) as its degradation is essential to the regulation of protein expression.^5^ RNA degradation is a major challenge for *in vitro* applications such as cell-free protein synthesis (CFPS), reverse transcription polymerase chain reaction (RT-PCR), quantitative RT-PCR (qRT-PCR), RNA-Seq and Northern Blot analysis, all of which rely on RNA integrity and purity.^5-8^ Maintaining the integrity of RNA molecules during storage is also a challenge, as complete removal/inactivation of RNases is difficult without damaging or denaturing the RNA sample or using toxic chemicals such as phenol and chloroform.

Techniques to mitigate RNA degradation *in vitro* have a long history. One prominent solution is the pretreatment of samples and solutions with diethylpyrocarbonate (DEPC), which is effective for ribonuclease inhibition.^9,10^ One issue with this solution, however, is that DEPC and other similar chemicals are known carcinogens and require caution and training for their use. These chemicals also react quite readily with amine, thiol, and alcohol groups and cannot be used in many biologic experiments where buffers and biologic reagents being used and produced often contain these side groups. DEPC can also alkylate RNA which renders it unusable for some applications.^11^ Biologically produced RNase inhibitors may also effectively inhibit ribonucleases, but their action is often specific to certain types of ribonucleases and they are often very expensive.^9,12,13^

One promising solution to some of these challenges is the use of inexpensive chemical (non-biologic) RNase inhibitors. Utilizing anionic polymers as a tool for RNase A inhibition is one chemical method that was initially tested over 50 years ago.^14,15^ More recently, it was reported that polyvinyl sulfonic acid (PVSA; average MW ∼2–5 kDa), a negatively charged polymer with sulfate branches, is a potent inhibitor of RNase A^16^. The repeating sulfate units resemble the repeating phosphate units that form the backbone of RNA and are thought to form competitive coulombic interactions with RNase A, thereby occupying its RNA-binding sites and effectively inhibiting RNase A.^16,17^

Here we describe experiments performed to assess the viability of PVSA beyond RNase A, as an inexpensive, safe, and effective inhibitor against bacterial RNases. We examine PVSA's effects in RNA stabilization in common *in vitro* applications, such as *in vitro* transcription (IVT) and coupled and decoupled *in vitro* transcription and translation. We further analyze the economic viability of using this polymeric RNase inhibitor. Our results suggest that certain applications, particularly RNA storage and *in vitro* transcription, can benefit from low-cost RNase inhibition through the use of PVSA.

## Results

### PVSA-mediated inhibition of RNase activity in bacterial lysate

**Figure 1.**
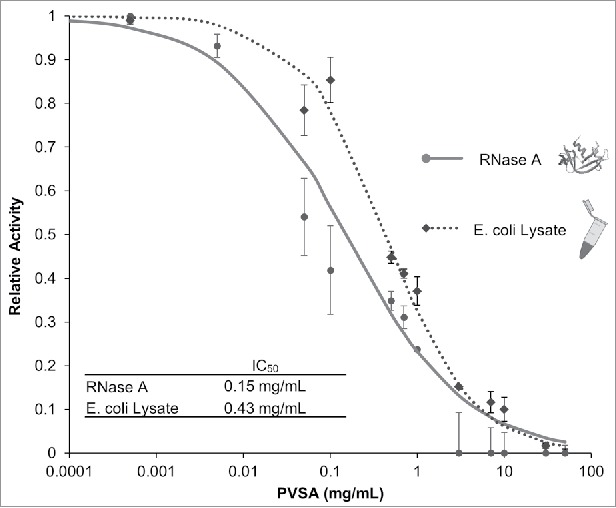
Inhibition of RNase Activity with PVSA. The relative RNase Activity of both RNase A and *E. coli* lysate was measured at varying concentrations of PVSA using RNaseAlert® (Ambion). The amount of PVSA required for 50% inhibition (IC_50_, inset) was determined from normalized data fit to a reciprocal semi-log response curve (n = 3, error bars represent 1 standard deviation).

To examine the RNase inhibitory potency of PVSA, we measured the ribonuclease activity of RNase A and *E. coli* lysate in the presence of PVSA. The assays were performed using Ambion's RNaseAlert® assay kit (IDT, IA, USA). Inhibition of RNase A (0.75 nM) was examined with increasing concentrations of PVSA (0.001 mg/mL – 50 mg/mL). Consistent with a previous report,^16^ PVSA effectively inhibited RNase A (Fig. 1; IC_50_ of 0.15 mg/mL PVSA with greater than 95% inhibition occurring at concentrations greater than 13 mg/mL of PVSA). We also tested the inhibition potency of PVSA against a bacterial lysate from *E. coli*, which contains diverse bacterial ribonucleases. Many of these intrinsic ribonucleases are known to escape inhibition from biologic RNase A inhibitors.^18-20^ The results showed significant inhibition of the lysate's combined RNase activities with an IC_50_ of 0.43 mg/mL PVSA (Fig. 1).

### Coupled *in vitro* transcription and translation

**Figure 2.**
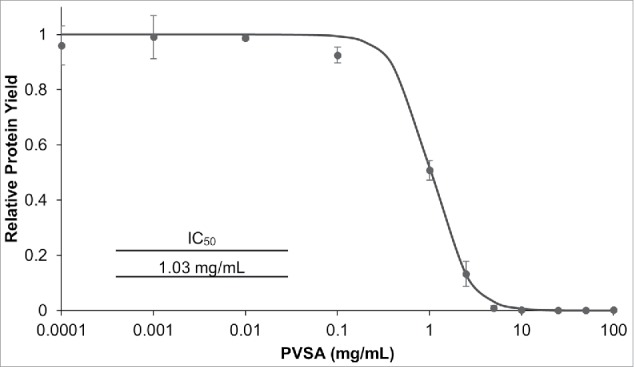
Inhibitory Effects of PVSA on Coupled *in vitro* Transcription and Translation Reactions. Varying concentrations of PVSA were added to an *E. coli*-based cell-free coupled transcription and translation system and GFP production yield is shown relative to production without PVSA. The amount of PVSA required for 50% inhibition (IC_50_, inset) was determined from normalized data fit to a reciprocal semi-log response curve (n = 3, error bars represent 1 standard deviation).

Next, PVSA's inhibitory capacities were explored in an *E. coli*-based cell-free protein synthesis (CFPS) reaction where transcription and translation were coupled. We introduced PVSA at varying concentrations to the coupled *in vitro* reaction and measured the total green fluorescent protein (GFP) synthesis by its fluorescence (Fig. 2). As increasing concentrations of PVSA were added, a strong inhibitory effect on protein synthesis was evident (IC_50_ value of 1.03 mg/mL) and essentially no protein synthesis was observed at 10 mg/mL PVSA.

### Decoupled *in vitro* transcription and translation

**Figure 3.**
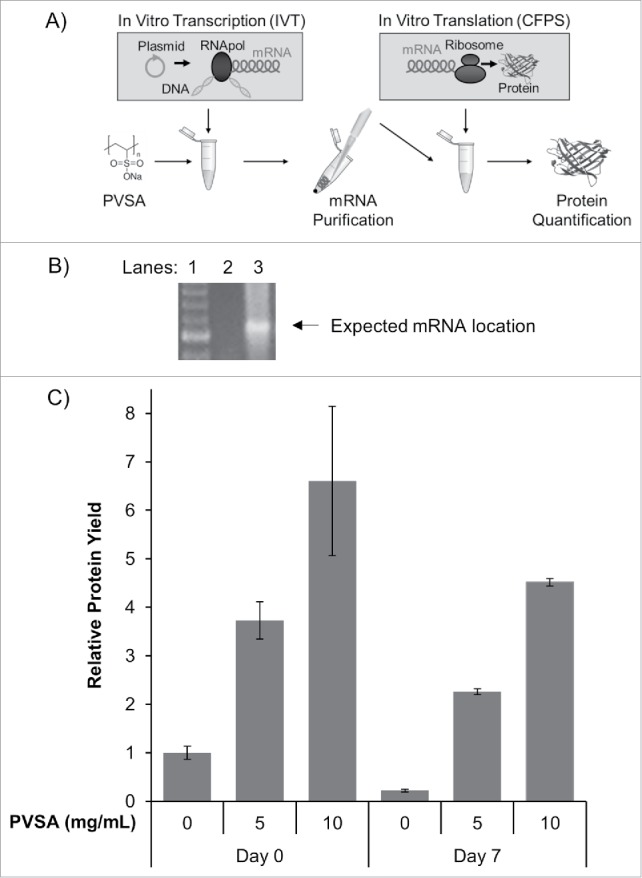
PVSA Effect on Decoupled *in vitro* Transcription with Subsequent Translation. (A) A schematic illustrates *in vitro* transcription (IVT) and subsequent purification with isopropanol precipitation and *in vitro* translation. (B) Image of mRNA product from IVT after agarose gel electrophoresis and staining with ethidium bromide. Lane 1 is the nucleic acid marker of double stranded DNA with bands corresponding to 400, 500, 600, 700, and 800 base pairs from bottom to top. Lane 2 is the IVT product where no PVSA was added. Lane 3 is the IVT product with 5 mg/mL PVSA. The expected migration location for the 898 nucleotide long mRNA is shown by arrow and corresponds to ∼600 base pairs of double stranded DNA due to the mRNA's single stranded and only partially hybridized nature. (C) Relative GFP protein yields as translated with mRNA produced by IVT in the presence of 0, 5, or 10 mg/mL PVSA and after 0 or 7 d of storage (n = 6, error bars represent one standard deviation).

To determine the basis of PVSA inhibition in the CFPS system, the processes of mRNA transcription and translation were decoupled (Fig. 3A). mRNA encoding GFP for subsequent translation was prepared in the presence of PVSA at varying concentrations by *in vitro* transcription (IVT) using the same plasmid (pY71-sfGFP) and RNA polymerase (T7 RNA polymerase) used in the coupled results above. An aliquot of these reactions was purified by precipitation with isopropanol, and the resuspended mRNA was assessed for storage stability and retained function. Gel electrophoresis suggests IVT reaction products stored for 7 d with 5 mg/mL PVSA had approximately 2 to 4 times the amount of mRNA as those without PVSA.

To determine whether the mRNA transcripts transcribed in the presence of PVSA were viable for translation, equal-volume aliquots of mRNA produced and purified from PVSA-treated and untreated IVT reactions were introduced into an *E. coli*-based cell-free translation system. The yield of GFP translated from the mRNA transcripts were normalized to the yield of GFP from mRNA produced without PVSA (Fig. 3C; Day 0, 0 mg/mL PVSA). This normalization facilitates comparison of relative protein yields as a response to increasing PVSA concentration and storage time.

The mRNA produced with PVSA had significantly better protein translation yields in the decoupled CFPS reaction than mRNA produced without PVSA. The protein yield from mRNA transcribed with 10 mg/mL PVSA resulted in a greater than 500% increase in protein translation when the mRNA was directly translated following transcription and purification (Fig. 3C). Storage of mRNA for 7 d at −20°C did result in a decrease in the protein synthesis levels; however, including 10 g/mL PVSA resulted in a 2,000% increase in protein production relative to mRNA transcribed without PVSA. These results suggest PVSA does not inhibit T7 RNA polymerase-mediated transcription, but likely inhibits a translation mechanism in the coupled transcription and translation system. Pretreatment of the IVT with PVSA and subsequent isopropanol precipitation does not appear to inhibit the RNA-programmed CFPS reaction and improves both mRNA and protein yield from the programmed lysate. Thus, PVSA appears to not co-precipitate with the RNA at significant concentrations during isopropanol precipitation and can be efficiently separated in this manner.

### Economic viability

We performed an economic analysis to determine the relative cost-effectiveness of PVSA as a ribonuclease inhibitor. The use of PVSA at 10 mg/mL for protection of mRNA during IVT is over 1,700 times less costly than using the manufacturer's recommended concentration for either the Murine RNase inhibitor® (NEB) or the Recombinant RNase inhibitor (Takara). For reference, using 10 mg/mL PVSA in place of the manufacturer's recommended concentration of Murine RNase inhibitor® or Recombinant RNase inhibitor reduces the cost of IVT reagents by more than 95%. Further, we compared the costs per mass of protein produced in a decoupled protein transcription and translation system with adding 10 mg/mL PVSA and without adding PVSA during transcription (Table 1). Using PVSA proved to be significantly less expensive (approximately 6-fold) on a cost basis per mg of protein produced. Estimated costs per mg protein with and without PVSA were $13 and $86 respectively (Table 1) as calculated using *in vitro* transcription/translation reagent costs published previously.^21^

**Table 1.** Economic Analysis of Decoupled Protein Synthesis with PVSA.

	Decoupled Protein Synthesis	
	Protein Yield (mg/mL)^a^	Estimated Cost/ mg Protein
With PVSA	0.409 +/− 0.095	$13
Without PVSA	0.062 +/− 0.009	$86

a Protein Yield +/− Standard Deviation (n=6)

## Discussion

### Coupled and decoupled protein synthesis

Biological protein-based RNase inhibitors typically evolved to target relatively small groups of RNases.^12^ For example, commercially available murine RNase inhibitor is known to specifically inhibit RNases A, B, and C, however it does not inhibit RNases T1 and H^20^. Thus multiple and diverse RNase inhibitors may be required for protection from multiple sources of RNases. Non-biologic inhibitors offer alternatives that have broader potential due to the lowered specificity of inhibitory interactions and reduced costs (Table 1). Although PVSA has been reported to be an inhibitor of RNase A activity, its activity against other RNases has not been previously reported.^14,16^ We have demonstrated that PVSA has inhibitory properties against RNase A as well as RNases in bacterial lysates (Fig. 1). Although this evidence is not exhaustive, the activity of PVSA against a spectrum of RNases suggests that its mechanism of inhibition allows it to be a potent inhibitor of several biologic ribonucleases with diverse catalytic mechanisms, substrate sequences and active sites.

The idea that PVSA can be an inhibitor of RNase A has previously been demonstrated, however, its applications for *in vitro* biologic systems were unclear.^16^ An increase of mRNA yield from IVT with PVSA and the resulting mRNA's viability for *in vitro* translation after isopropanol precipitation was demonstrated in this work. As mentioned, however, a limitation of PVSA use was shown in its inhibition of a coupled cell-free protein synthesis reaction. Such a deleterious effect could be explained by PVSA interfering with one or more mechanisms of translation. This is especially likely considering PVSA's hypothesized mechanism of inhibition where PVSA's polyanionic nature in solution causes it to resemble a ribonucleic acid and competitively bind to RNases.^16^ As more PVSA binds with the ribonuclease, less RNA would be degraded, which allows for a higher relative yield of mRNA. However, the RNA-mimicking structure of PVSA could also inhibit the ribosome and other RNA-binding proteins in addition to RNases. Interestingly, PVSA does not inhibit T7 RNA polymerase during IVT, suggesting that the inhibitory properties of PVSA are not generalizable to all nucleic acid-protein interactions and PVSA does not likely mimic double-stranded DNA. It is worth noting that another chemical RNase inhibitor, aurin tricarboxylic acid (ATA) inhibits interactions between RNA polymerase and its DNA template.^22^ Further, ATA binds both RNA and DNA, can partition into the insoluble fraction in an alcohol precipitation, and can be only partially removed by size-exclusion chromatography.^23^ Therefore, PVSA occupies a different niche from ATA in applications of RNase inhibitors as PVSA appears to be readily separable from nucleic acids by alcohol precipitation. It is thought that the relatively small molecular weight of PVSA (2 to 5 kDa) compared with that of mRNA (about 35 kDa in this work) permits it to solubilize effectively in alcohol. It is also thought that the charge to mass ratio of PVSA, about 2.5 times greater than that of RNA also plays a role in permitting solubilization. These unique qualities that facilitate PVSA separation by alcohol precipitation could potentially be exploited to separate PVSA by ion exchange chromatography which is commonly used to purify nucleic acids. This evidence supports the judicious use of PVSA in IVT systems, where follow-on enzymatic treatment of RNA after production (*e.g.*, translation, end-labeling or ligation) could be performed after removal of PVSA, but is contraindicated in those systems that rely on translation or other RNA-binding proteins.

### RNA storage

A comparison of relative yields of protein at different time points (Fig. 3) suggests that addition of PVSA can enhance preservation of viable mRNA transcript under −20°C storage conditions. Currently, one common method of storing RNA involves solubilizing RNA in water treated with diethylpyrocarbonate (DEPC).^24^ However, as discussed previously, DEPC and similar chemicals are known carcinogens, are unable to treat certain buffers that may contain amine, thiol, or alcohol groups and cannot be directly added to a reaction solution without side-reactions,^10^ including modification of RNA.^11^ Results from our decoupled translation system demonstrated that for samples containing no PVSA, relative yields at 2 time points (days 0 and 7) were similar, indicating similar RNA recovery, with significantly better RNA recovery at concentrations of 5–10 mg/mL of PVSA. Such evidence suggests PVSA may act as a potent inhibitor of ribonucleases to protect mRNA degradation in storage conditions.

## Materials and methods

### Inhibition of RNases

Ambion's RNaseAlert® kit (Catalog number: AM1964) comprising a double-labeled oligoribonucleotide with both fluorescent and quenching moieties was used as a substrate for treatment with RNases in the presence or absence of Polyvinyl Sulfonic Acid (PVSA; average MW ∼2–5 kDa, Sigma Aldrich, Catalog Number: 278424) as a ribonuclease inhibitor. Cleavage of this RNA derivative substrate molecule separates the fluorophore from the quenching moiety and enables detection of fluorescence, as monitored with a Biotek® Synergy MX plate reader spectrophotometer in fluorimetry mode using Corning® 96 well black plates. Excitation and emission peaks were measured at 480/520 nm respectively. Relative activity for both RNase A and RNase activity in bacterial lysate were measured at varying concentrations of PVSA. The data were normalized and fit to a reciprocal semi-log response curve to determine IC_50_ values (concentration of inhibitor required to inhibit the reaction by 50%).

Bacterial lysate was prepared as described previously^25-27^ using the *Escherichia coli* B strain BL21 Star™ (DE3) (Life Technologies C601003; BL21 (DE3) Star™ is *rne131* F^−^ *ompT gal dcm lon hsdS_B_*(*r_B_*^−^*m_B_*^−^) λ(DE3 [*lacI lacUV5-T7p07 ind1 sam7 nin5*]) [*malB*^+^]_K-12_ (λ^S^)). The bacterial cells were grown, harvested, and lysed using an Avestin EmulsiFlex® B-15 Homogenizer with 3 passes at 21,000 psi. The lysate was clarified by centrifugation at 16,000 g, 4°C for 30 min and the supernatant was collected, aliquotted, flash frozen, and stored at −80°C until use. The clarified lysate was then added to the RNaseAlert® reaction at a 0.1% (v/v) concentration. Ribonuclease A (Sigma Aldrich, Catalog Number: R6513) was added to 0.75 nM.

### *In vitro* transcription (IVT)

*In vitro* Transcription (IVT) reactions were prepared with varying concentrations of PVSA. Reactions were prepared with nucleotide triphosphate mixture containing 2 mM each ATP, GTP, CTP, and UTP (Sigma Aldrich catalog numbers A2383, G8877, 30320, 94370, respectively), T7 RNA polymerase buffer containing 50 mM Tris-Cl (Sigma Aldrich RES3098T-B7) at a pH of 7.5, 15 mM MgCl_2_ (as hexahydrate; Sigma Aldrich 1374248), 5 mM dithiothreitol (Bachem USA, Q-1225), and 2 mM spermidine (Sigma Aldrich S2626). A mixture of 3 mM spermidine and 2 mM putrecine (Sigma Aldrich P5780) was also used in the reaction. T7 RNA polymerase enzyme was prepared as described previously^28^ and added at 2% v/v. PVSA was added at concentrations of 0, 5, and 10 mg/mL.

The 550 µL IVT reactions were templated using the 50 µg/mL pY71-sfGFP plasmid ^29^ and incubated for 90 minutes at 37°C with vigorous agitation (200 rpm). Separate aliquots were then purified and resuspended in 55 µL RNase free water using an isopropanol precipitation procedure ^30^ or placed in storage (−20°C, 7 days) until precipitation. Aliquots of the IVT reaction after 7 d of storage and after purification by isopropanol precipitation were imaged with electrophoreses on a 1% agarose gel stained with ethidium bromide using a Tris-Acetate-EDTA (TAE) buffer (166 V constant, 1 hour). 1Kb Plus DNA Ladder (Invitrogen) was used as a nucleic acid reference. The gel was then imaged using a Fluor-S BioRad Multiimager with UV filter to visualize mRNA bands (898 nt). Densitometry was performed to estimate the relative amount of mRNA.

Analysis of reagent costs were determined with the prices listed by Sigma Aldrich, Bachem, NEB, and Takara and calculated using other *in vitro* transcription/translation reagent costs published previously.^21^ In comparing the cost of using Murine RNase inhibitor® (NEB) or Recombinant RNase inhibitor (Takara), the manufacturers' recommendation of 1,000 unit/mL for each was applied.

### Decoupled *in vitro* transcription and translation

The mRNA transcripts, produced by IVT and purified by precipitation as described above, were then added in equal volume (15 µL) aliquots to an *E. coli*-based cell-free translation PANOxSP system (150 µL final volume). The cell-free reactions were then performed as described previously with the exception that plasmid DNA was not added to the reaction.^25-27^ Relative GFP protein yields were determined using a Biotek Synergy MX plate reader spectrophotometer in fluorimetry mode to measure emission at 510 nm (excitation at 485 nm). Excitation data were normalized to the sample with no PVSA added (Day 0 sample with 0 mg/mL PVSA).

### Coupled *in vitro* transcription and translation (cell-free protein synthesis)

*E. coli*-based cell-free protein synthesis reactions based on the PANOxSP were performed as described previously^25-27^ and using the same reagents used in the decoupled *in vitro* translation system described above. As an exception the pY71-sfGFP plasmid, which is transcribed by T7 RNA polymerase in concert with translation, was added instead of adding mRNA transcribed by T7 RNA polymerase from pY71-sfGFP in a prior IVT reaction. The coupled CFPS reaction without PVSA was compared with the coupled system where PVSA was added at varying concentrations (0.0001–100 mg/ml) and the yield was normalized to GFP production without PVSA. Protein production yields of GFP were determined with fluorescence as described above.

## Conclusion

Here we demonstrate the potential of PVSA as a low cost chemical alternative to biologic RNase inhibitors and present data suggesting its ability to inhibit RNases beyond RNase A. While PVSA is 1,700-fold less expensive and was shown to successfully preserve mRNA, PVSA appears to inhibit mechanisms that involve RNA binding such as translation. To overcome this limitation, the successful removal of PVSA using a simple alcohol precipitation mechanism is reported. It is important to note that this is a proof-of-concept study demonstrating the potential of the chemical RNase inhibitor PVSA, and more head-to-head studies comparing PVSA directly with biologic RNase inhibitors is needed before widespread use. However, here we further report the potential of chemical inhibitors such as PVSA to drastically decrease the costs and complexity of preserving RNA in an RNase ubiquitous world.
